# Inhibition in the Human Auditory Cortex

**DOI:** 10.1371/journal.pone.0155972

**Published:** 2016-05-24

**Authors:** Koji Inui, Kei Nakagawa, Makoto Nishihara, Eishi Motomura, Ryusuke Kakigi

**Affiliations:** 1 Department of Integrative Physiology, National Institute for Physiological Sciences, Japan; 2 Multidisciplinary Pain Center, Aichi Medical University, Japan; 3 Department of Neuropsychiatry, Mie University Graduate School of Medicine, Japan; Chiba University Center for Forensic Mental Health, JAPAN

## Abstract

Despite their indispensable roles in sensory processing, little is known about inhibitory interneurons in humans. Inhibitory postsynaptic potentials cannot be recorded non-invasively, at least in a pure form, in humans. We herein sought to clarify whether prepulse inhibition (PPI) in the auditory cortex reflected inhibition via interneurons using magnetoencephalography. An abrupt increase in sound pressure by 10 dB in a continuous sound was used to evoke the test response, and PPI was observed by inserting a weak (5 dB increase for 1 ms) prepulse. The time course of the inhibition evaluated by prepulses presented at 10–800 ms before the test stimulus showed at least two temporally distinct inhibitions peaking at approximately 20–60 and 600 ms that presumably reflected IPSPs by fast spiking, parvalbumin-positive cells and somatostatin-positive, Martinotti cells, respectively. In another experiment, we confirmed that the degree of the inhibition depended on the strength of the prepulse, but not on the amplitude of the prepulse-evoked cortical response, indicating that the prepulse-evoked excitatory response and prepulse-evoked inhibition reflected activation in two different pathways. Although many diseases such as schizophrenia may involve deficits in the inhibitory system, we do not have appropriate methods to evaluate them; therefore, the easy and non-invasive method described herein may be clinically useful.

## Introduction

Cortical neural circuits are composed of excitatory pyramidal cells (PCs) and inhibitory interneurons. In order to understand how circuits process information, their functions as well as the balance between them have to be elucidated. However, the roles of inhibitory interneurons have not been examined in as much detail as PCs. One reason is their great diversity in morphology, connections, intrinsic electrophysiological properties such as firing, synaptic dynamics including their excitatory inputs, subcellular domains targeting, and neuropeptide expression [1–9]; therefore, it is difficult to understand their function in a systematic manner. Another reason may be the challenges associated with observing the activity of interneurons *in vivo*. Therefore, their actual function in a circuit largely remains unknown. Information on cortical inhibition in humans is very limited. Since many diseases including epilepsy [10,11], schizophrenia [12], depression [13], bipolar affective disorder [14], panic disorder [15], autism [16], and essential tremor [17] may have involve in inhibition, non-invasive methods to evaluate the functions of inhibitory circuits are desired.

Inhibitory postsynaptic potentials (IPSPs) cannot be recorded non-invasively, at least in a pure form, which is an important problem, particularly in humans. As a candidate for an indirect method to measure inhibitory processes, we recently developed a technique by which to observe prepulse inhibition (PPI) in the human cortex [18,19]. Conventional PPI is a phenomenon in which a weak leading sensory stimulus (prepulse) suppresses startle responses evoked by a strong sensory stimulus [20,21]. An intense sound is typically used to evoke startle reflexes. The blink reflex is measured in humans by electromyography and whole-body flinching is measured in rodents using stabilimeter chambers. Although PPI has the merit of findings being comparable between humans and experimental animals because it is common across mammals [22], the pathway is complicated and its mechanisms are largely unknown [23]. In our paradigm, change-related cortical responses [24] are used as an index instead of motor responses. The change-related cortical response is a sensory-evoked cortical activation that is specific to a change in a stimulus, and is recorded very clearly using electroencephalography (EEG) or magnetoencephalography (MEG) with a weak change stimulus that evokes no startle reflex. Similar to the PPI of startle reflexes [25], the change-related cortical response was previously shown to be clearly suppressed by a prior weak change stimulus that itself evokes only a weak or no cortical response [18,19]. The change-related auditory response is known to provide high test-retest reliability with a coefficient r around 0.9 [18,26,27]. Therefore, we may be able to observe the activity of interneurons in a specific circuit using the PPI of the auditory change-related cortical response. PPI is considered to be a sensory gating process by which sensory information is screened so that an individual can focus on the most salient events. Gating is one of the specific goals of interneurons [28].

In the present study, we sought to clarify whether the PPI of change-related cortical responses represented an active inhibitory process, and also whether the time course of the inhibition was consistent with those of IPSPs. For the former, we examined whether the magnitude of inhibition depended on the magnitude of prepulse-evoked brain responses. If suppression of the test response depends on events in excitatory synaptic transmission exclusively, the degree of inhibition should depend on the magnitude of prepulse-evoked excitatory responses. For the latter, we employed a paired-pulse stimulation paradigm with various conditioning-test intervals from 10 to 800 ms. By manipulating the timing of the prepulse, we can estimate the time course of inhibitory activity of the prepulse. The time course of IPSPs is well known in whole-cell patch clamp studies consisting of an early component peaking at 20–30 ms (e.g. [29]) and a late one peaking at 80–200 ms (e.g. [30]). In addition, very late IPSPs are evoked at 250–500 ms when a train of presynaptic action potentials is applied [31]. Therefore, by comparing the time course of these IPSPs and that of PPI, we could know whether the present inhibition was consistent with IPSP-induced modulation. The results obtained supported these hypotheses; therefore, the PPI of the change-related response may be useful for non-invasive assessments of the inhibitory functions of interneurons in individuals.

## Methods

This study was approved in advance by the Ethics Committee of the National Institute for Physiological Sciences, Okazaki, Japan, and written consent was obtained from all subjects. The experiment was performed on 13 (three females and ten males) healthy volunteers, aged 24–51 (35.2 ± 8.7) years. They were asked to refrain from alcohol, caffeine, and smoking for at least 12 hours prior to the experiment. There were three smokers. None of the subjects had any history of mental or neurological disorders or substance abuse in the last two years. They were free of medication at testing. They had a hearing threshold lower than 30 dB at 1000 Hz as assessed by an audiometer (AA-71, Rion, Tokyo, Japan).

### Auditory stimuli

**Fig 1:**
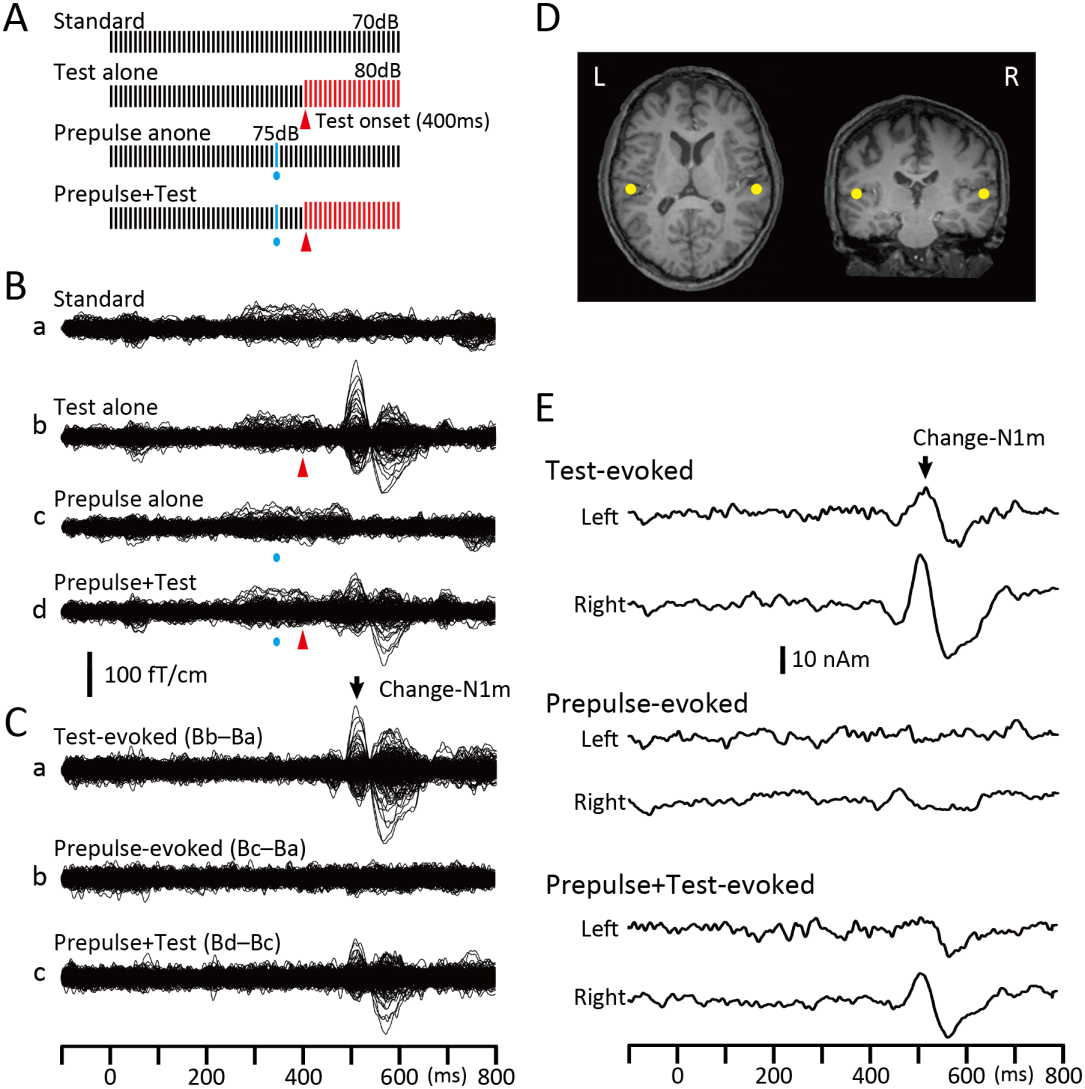
The change-related cortical response and its inhibition by a weak prepulse. (A) Sound stimuli consisted of a train of 1-ms clicks at 100 Hz in repetitive frequency and 70 dB SPL in sound pressure. An abrupt increase in sound pressure of 10 dB evoked the Test response, while that for the prepulse to inhibit the Test response was 5 dB from the background (Standard). Each bar indicates a single click. (B) Superimposed MEG waveforms recorded from the 204 channels following the four sound stimuli in a single subject, Standard (a), Test alone (b), Prepulse alone presented 60 ms before the Test onset (c), and Prepulse + Test (d). (C) Difference waveforms used to analyze the data. The Test alone response was obtained by subtracting Standard-evoked waveforms (Ba) from Test-evoked waveforms (Bb). The Prepulse alone response was obtained by subtracting Standard-evoked waveforms (Ba) from Prepulse-evoked waveforms (Bc). The Prepulse + Test response was obtained by subtracting Prepulse-evoked waveforms (Bc) from waveforms for the Prepulse + Test stimulus (Bd). (D) The dipole locations estimated at approximately the peak latency of Change-N1m for the Test-evoked response (back arrow) were superimposed on each subject’s MR images. (E) Source strength waveforms obtained by applying the dipole model in D to the difference waveforms in C.

Repeats of a 1-ms sine wave click at 100 Hz [19] were used for all experiments. There were four types of sound stimuli (Fig 1A): repeats of the same click at 70 dB sound pressure level (SPL) (Standard), repeats of standard clicks followed by 20 clicks of 80 dB (Test alone), the Test preceded by a click of 75 dB (Prepulse + Test), and the Standard with a Prepulse (Prepulse alone). Sound stimuli were presented binaurally through ear pieces (E-A-Rtone 3A, Aero Company, Indianapolis, IN).

### MEG recordings

Magnetic signals were recorded using a 306-channel whole-head type MEG system (Vector-view, ELEKTA Neuromag, Helsinki, Finland), which comprised 102 identical triple sensor elements. Each sensor element consisted of two orthogonal planar gradiometers and one magnetometer coupled to a multi-superconducting quantum interference device (SQUID), and thus provided 3 independent measurements of the magnetic fields. In the present study, we analyzed MEG signals recorded from 204 planar-type gradiometers. These planar gradiometers are sufficiently powerful to detect the largest signal just over local cerebral sources. Signals were recorded with a bandpass filter of 0.1–300 Hz and digitized at 1004 Hz. An analysis was conducted from 100 ms before to 300 ms after the onset of the Test (Test-evoked response) or the onset of the Prepulse (Prepulse-evoked). Epochs with MEG signals larger than 2.7 pT / cm were rejected from the averaging.

### Procedures

Experiments were conducted in a quiet, magnetically shielded room. Subjects sat in a chair and watched a silent movie on a screen 1.5 m in front of them throughout the experiments. Five experiments: Experiments 1–1 ~ 1–4 and Experiment 2, were carried out on different days. In all experiments except Experiment 1–3, all 13 subjects were tested. Four subjects did not participate in Experiment 1–3 because of their personal circumstances.

#### Experiment 1–1

The effects of the Prepulse-Test interval were examined from 10 to 200 ms. The Standard sound was 600 ms in length. The Prepulse was presented either 10, 30, 60, 100, 150, or 200 ms before the Test presented at 400 ms in the click train. Therefore, there were 14 stimuli: 1) Standard, 2) Test alone, 3)–8) Prepulse alone, and 9)–14) Prepulse + Test. The fourteen stimuli were presented randomly at an even probability at a trial-trial interval of 1000 ms. A total of 120–125 artifact-free epochs were averaged for each stimulus.

Recorded MEG waveforms were subjected to band-pass filtering of 1–75 Hz and analyzed as previously reported [18,19]. In brief, the Test-evoked response (Fig 1Ca) was obtained by subtracting the waveform for the Standard (Ba) from that for the Test alone stimulus (Bb). Similarly, the Prepulse + Test response (Cc) was obtained by subtracting the waveform for the Prepulse alone stimulus (Bc) from that for the Prepulse + Test stimulus (Bd). Using the subtracted Test-evoked response waveform, an equivalent current dipole for the magnetic component at approximately 130 ms, Change-N1m, was estimated for each hemisphere using BESA (NeuroScan, Mclean, VA)(Fig 1D). The two-dipole model obtained was applied to all subtracted waveforms, and the source strength waveform (Fig 1E) was used to measure the amplitude of Change-N1m. The same procedures were applied for Experiment 1–4 and Experiment 2.

#### Experiment 1–2

**Fig 2:**
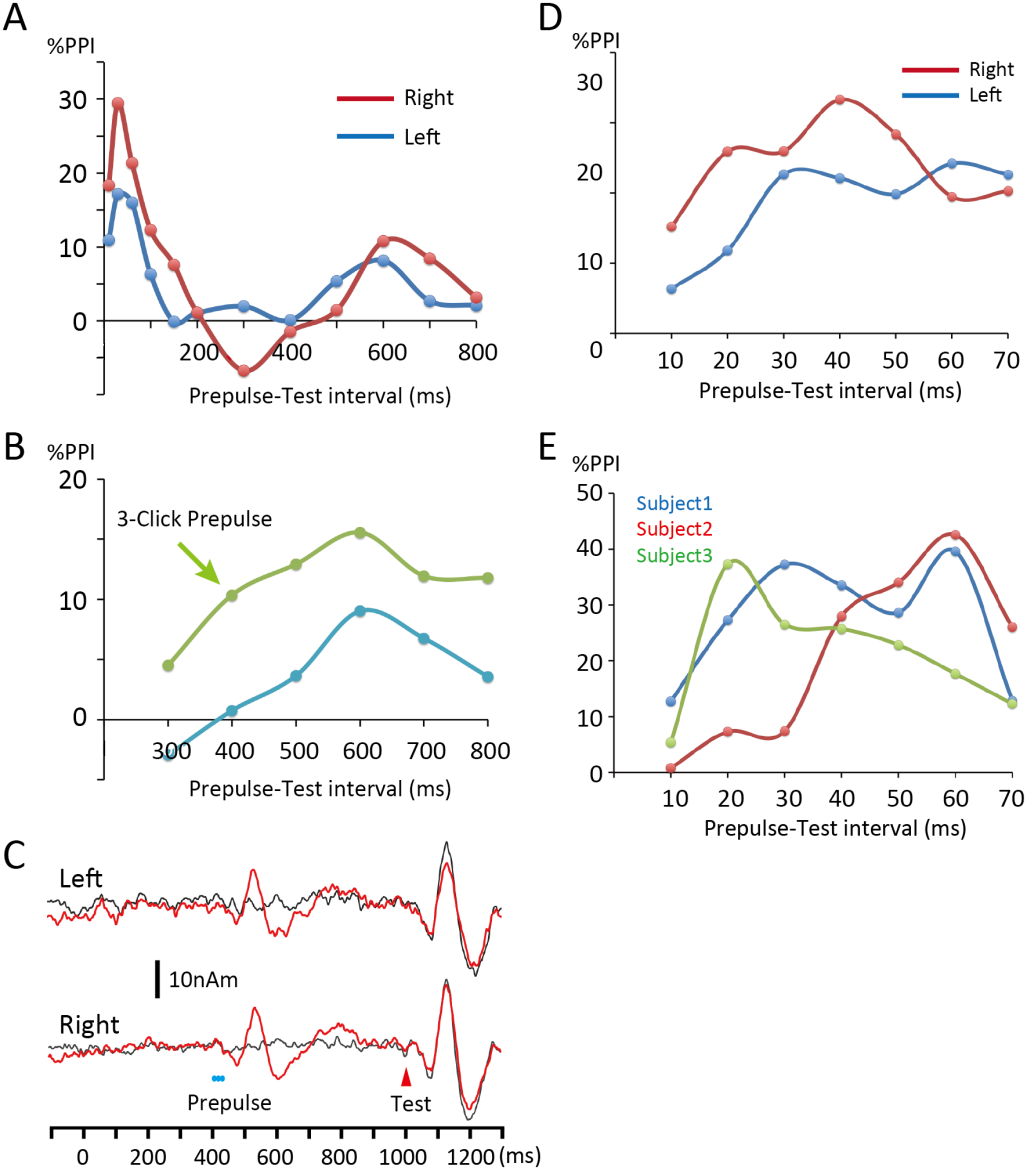
Time course of inhibition. (A) The results of Experiment 1–1 (10–200 ms) and Experiment 1–2 (300–800 ms) are shown together. (B) Comparison of the time course of long-latency inhibition between a prepulse of a single click (Experiment 1–2, blue line) and that of 3 clicks (Experiment 1–3, green line). Average values between hemispheres are shown (n = 9 subjects). Although the 3-click Prepulse confirmed the time course of the inhibition by the weak prepulse in Experiment 1–2, inhibition was still weak. (C) Waveforms show the Test alone (black) and Test + Prepulse response (red) for the 600ms-Prepulse condition in Experiment 1–3. (D) Results of Experiment 1–4. Although inhibition was the greatest for the 40ms-Prepulse on average, the inhibition curve was biphasic in both hemispheres. (E) The time course of the short-latency inhibition in three representative subjects showing two peaks at 30 and 60 ms (Subject 1), and single peaks at 60 ms (Subject 2) and at 20 ms (Subject 3). %PPI, percent prepulse inhibition.

The effects of longer Prepulse-Test intervals were explored using a 75-dB click (Prepulse) presented either 300, 400, 500, 600, 700, or 800 ms before the Test at 1000 ms in the click train. The Standard sound was 1200 ms in length. Unlike the shorter intervals in Experiment 1–1, responses to the Test and Prepulses did not temporally overlap in this recording. In addition, compared to Experiment 1–1, a markedly longer time was needed to complete the recording. Therefore, in Experiments 1–2 and 1–3, there was only one Prepulse-alone condition, in which a prepulse was presented 400 ms before the Test. The Prepulse + Test response were evaluated without subtraction procedures (Fig 2C) as described elsewhere [19]. Therefore, there were nine sound stimuli: 1) Standard, 2) Test alone, 3) Prepulse alone at 400 ms, and 4)–9) Prepulse + Test. The nine stimuli were presented randomly at an even probability at a trial-trial interval of 1600 ms. A total of 120–125 artifact-free epochs were averaged for each stimulus.

#### Experiment 1–3

Since the results of Experiment 1–2 showed significant inhibition for the Prepulse at 600 ms only, we considered it necessary to confirm the inhibition itself and the time course of the inhibition further using stronger prepulses. All the procedures in Experiment 1–3 were the same as those in Experiment 1–2, except that the prepulse used was three consecutive clicks. For example, the Prepulse inserted 600 ms before the Test was composed of three clicks of 75 dB at 600, 590, and 580 ms before the Test (Fig 2C). It was previously shown that IPSPs at these latencies were sensitive to the numbers of action potentials (APs) in presynaptic PCs [31].

#### Experiment 1–4

Short-latency inhibition was examined in more detail using prepulses presented either 10, 20, 30, 40, 50, 60, or 70 ms before the Test. Other than the prepulse timing, the procedures of recordings and analyses were similar to those in Experiment 1–1.

#### Experiment 2

**Fig 3:**
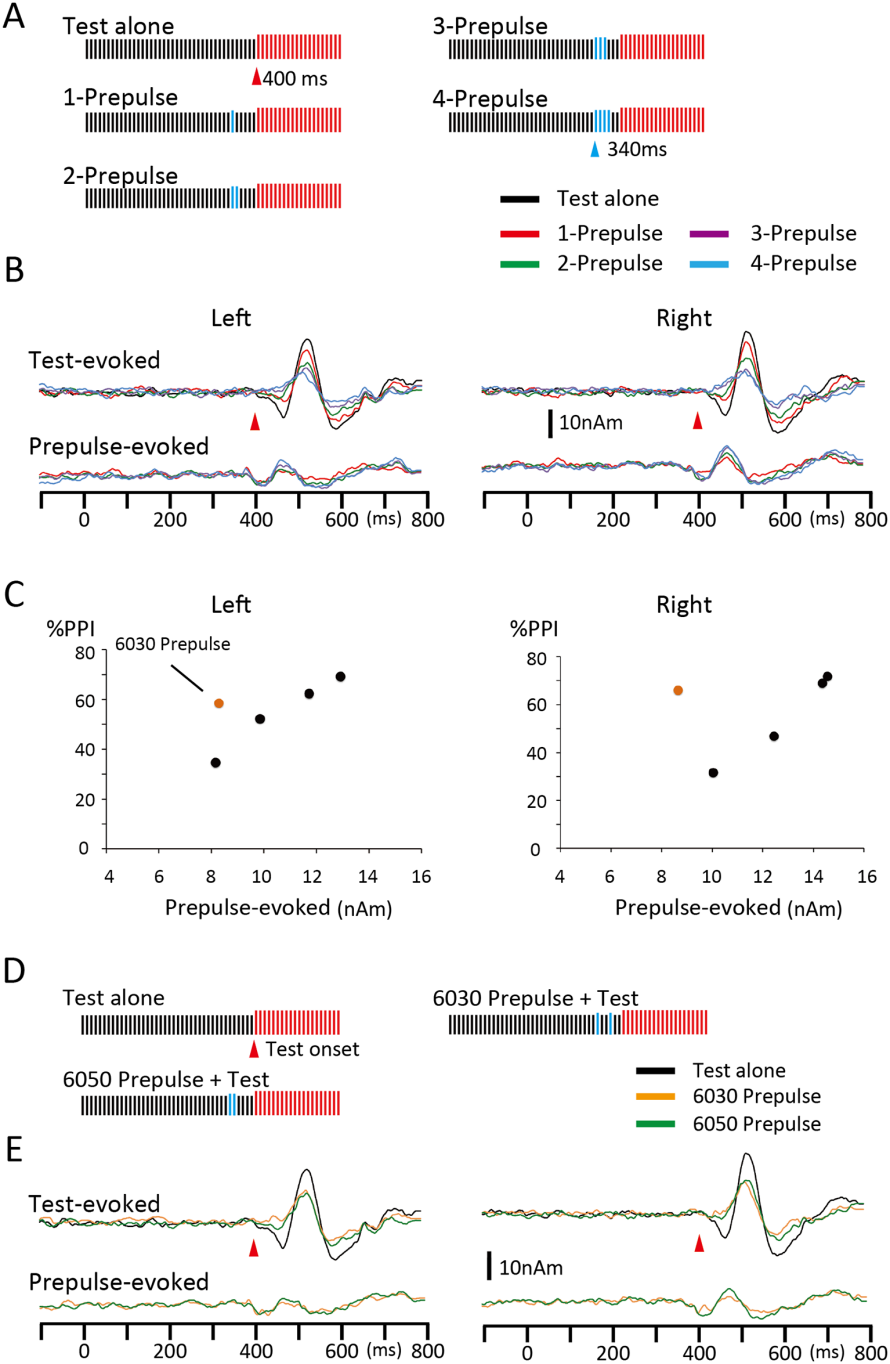
Effects of prepulses with a click train. (A) The magnitude of inhibition and Prepulse-evoked excitatory responses were compared among four Prepulses, with a single 75-dB click 60 ms (1-Prepulse), two clicks 60 and 50 ms (2-Prepulse), three clicks 60, 50, and 40 ms (3-Prepulse), and four clicks 60–30 ms (4-Prepulse) before the Test onset. (B) Grand-averaged Test- and Prepulse-evoked responses across 13 subjects. (C) Black filled circles are plots of the degree of inhibition (y-axis, percent inhibition) against the amplitude of the Prepulse-evoked response (x-axis) for the four Prepulses. Orange filled circles show data for the 2-click prepulse in D and E. (D) Comparison of excitatory and inhibitory effects of two Prepulses with two clicks at 60 and 30 ms (6030 Prepulse) and 60 and 50 ms (6050 Prepulse identical with 2-Prepulse in A). (E) Grand-averaged waveforms of Test- and Prepulse-evoked responses for Test alone (black line) and with 6050 (green) or 6030 Prepulse (orange) conditions. The excitatory effect (Prepulse-evoked) was stronger for the 6050 Prepulse, whereas the inhibitory effect was stronger for the 6030 Prepulse.

The effects of prepulses with a train of clicks were examined. There were five prepulses: a click of 75 dB presented at 60 ms before the onset of the Test (1-Prepulse), two clicks at 60 and 50 ms (2-Prepulse), three clicks at 60, 50, and 40 ms (3-Prepulse), four clicks at 60, 50, 40, and 30 ms (4-Prepulse), and two clicks at 60 and 30 ms (6030 Prepulse) (Fig 3A and 3D). The Test and Standard were the same as those in Experiment 1. Therefore, there were 12 stimuli: Standard, Test alone, five Prepulse alone, and five Prepulse + Test. Epochs of 120 ~ 125 trials were averaged. This experiment was carried out to confirm that PPI was not an event within PC-PC excitatory transmission. Since the results of Experiment 1 showed that the degree of inhibition largely depended on the timing of the Prepulse, we considered that inhibition would be strong regardless of the magnitude of the Prepulse-evoked response when inhibitory activities caused by each prepulse click are effectively summated.

### Analysis

The peak amplitude of Change-N1m was measured between the peak in Change-N1m within the period of 100–200 ms and the peak of the polarity-reversed earlier component within 50–100 ms [18,32]. The percent inhibition of the Change-N1m amplitude by the Prepulse (%PPI) was defined as (Test alone response–(Prepulse + Test response)) / Test alone response * 100. The Change-N1m amplitude was compared among Test-evoked responses using a two-way repeated measures ANOVA with Prepulse (without and with prepulses at 10, 30, 60, 100, 150, and 200 ms, for example in Experiment 1–1) and Hemisphere as the independent variables. To compare the difference between conditions, post-hoc multiple comparisons were done with Bonferroni-adjusted t-tests. All statistical analyses were performed at the 0.05 level of significance. When the sphericity assumption was violated, the Greenhouse–Geisser correction coefficient epsilon was used for correcting the degrees of freedom and then the F-value and significance probability were re-calculated. Data are expressed as the mean ± standard deviation (SD).

## Results

### Experiment 1

The test stimulus (Test), an abrupt increase in sound pressure by 10 dB in a continuous click train at 100 Hz and 70-dB SPL (Fig 1A), evoked a clear response at approximately 130 ms (Change-N1m) that was preceded by a polarity-reversed earlier component at approximately 60 ms (Change-P50m) [33]. As previously reported [32–34], the main cortical source for Change-N1m was estimated to be located in the lateral part of the transverse gyrus or superior temporal gyrus, which are lateral to the primary auditory cortex. Based on its temporal dynamics and activated location, Change-N1m reflects higher stages following a series of feedforward primary processes [35]. Although additional sources in other cortical areas such as the planum temporale appeared to significantly improve the goodness-of-fit of the model in some subjects [32,35], the two-dipole model was used in the present study in order to simplify the data interpretation. Although this might affect the results, our preliminary analyses using a multi-dipole model showed that PPI was not markedly different among cortical sources. Fig 1E shows an example of Change-N1m and its inhibition by a prepulse presented 60 ms before the Test stimulus.

In Experiment 1–1, the effects of the Prepulse-Test interval were examined in order to explore the time course of inhibition by inserting the Prepulse 10, 30, 60, 100, 150, and 200 ms before the Test onset. The results of a two-way ANOVA (Hemisphere 2 X Prepulse 7) showed that the Prepulse was a significant factor for determining the amplitude of the Test response (F_6,72_ = 13.06, p = 6.2 x 10^−10^, partial η^2^ = 0.52), but Hemisphere was not (F_1,12_ = 0.02, p = 0.97). Fig 2A shows scatter plots of the percent prepulse inhibition (%PPI) against the Prepulse-Test interval. The %PPI was largest for the Prepulse 30 ms prior to the Test onset (30ms-Prepulse) in both the left (% PPI = 17.2%) and right (29.5%) hemispheres. Bonferroni’s post hoc tests showed that the Test + Prepulse response was significantly smaller for the 10ms- (p = 0.007), 30ms- (< 0.0001), and 60ms-Prepulse (0.006) than the Test alone response. Furthermore, inhibition was significantly stronger for the 30ms-Prepulse than the 10ms-Prepulse (p = 0.037).

We further explored a longer time range in Experiment 1–2 by manipulating the Prepulse-Test interval from 300 to 800 ms. Although the Change-N1m amplitude differed significantly among the seven conditions (F_6, 72_ = 4.06, p = 0.0015, partial η^2^ = 0.25), inhibition was weaker than that of the shorter intervals in Experiment 1–1 with a significantly smaller response than the Test response only being observed for Prepulse 600 ms before the Test (p = 0.008) (Fig 2A). No significant difference was noted between hemispheres (F_1,12_ = 0.093, p = 0.77). This result suggests that the long-latency inhibition required a stronger prepulse. By considering the possibility that, in some subjects, the effects of a single click prepulse did not reach the threshold needed to exert an inhibitory action, we further tested a prepulse with a train of three clicks (Experiment 1–3). Although the train prepulse augmented inhibition and confirmed the time course of the long-latency inhibition (Fig 2B, green line), inhibition was still weaker than that of a similar train prepulse for the short latency inhibition (> 60% PPI), as described in Experiment 2. Fig 2C shows the grand-averaged waveforms for the 600ms-Prepulse (3-click) across nine subjects with a strong Prepulse-evoked response, but modest inhibition.

We next examined the short-latency inhibition in more detail using 10–70 ms Prepulses in a 10-ms step (Experiment 1–4). Inhibition was the greatest on average for the 40ms-Prepulse (27.7%), but did not differ significantly among the 20–70 ms-prepulses (p > 0.87). In both hemispheres, the time course of inhibition did not show a single peak (Fig 2D), implying overlapping multiple sources for inhibition. To support this notion, an approximately inverted-U-shaped time course peaking at 40 ms was only observed in four hemispheres out of 26. The time course showed a peak at 20–30 ms (Subject 3 in Fig 2E, n = 4 hemispheres) or at 50–60 ms (Subject 2, n = 7). In the remaining 11 hemispheres, the inhibition time course was biphasic with peaks at 20–30 and 50–60 ms (Subject 1). These results suggested that at least two sources with distinct time courses were responsible for the short-latency inhibition.

The peak latency and amplitude of the Test- and Prepulse-evoked responses are listed in Tables 1 and 2. Prepulse-evoked responses were weak. For example, when the response amplitude was expressed as a multiple of the standard deviation (SD) of the prestimulus baseline, the amplitude of the 30ms-Prepulse-evoked response in Experiment 1–1 was 4.1 ± 2.4 SD for the left hemisphere and 5.5 ± 2.5 SD for the right. Since the amplitude in the present study was a peak-to-peak measurement, these values were the lower limit to consider the response to be significant. The results of an ANOVA (Hemisphere 2 X Prepulse 6) revealed that neither the timing of the Prepulse (F_5,60_ = 0.85, p = 0.52) nor Hemisphere (F_1,12_ = 0.05, p = 0.83) significantly affected the amplitude of the Prepulse-evoked response in Experiment 1–1. Therefore, there was no clear relationship between the amplitude of the Prepulse-evoked response and %PPI (r = -0.11 ± 0.6 and 0.34 ± 0.35 for the left and right hemispheres, respectively). Similarly, the amplitude of the Prepulse-evoked response did not differ significantly among the seven prepulse conditions (F_6,72_ = 1.12, p = 0.36) or between hemispheres (F_1,12_ = 0.28, p = 0.61) in Experiment 1–4. The correlation coefficient r between the Prepulse-evoked amplitude and %PPI was 0.03 ± 0.48 for the left hemisphere and 0.21 ± 0.4 for the right.

**Table 1:** 

**Experiment 1–1**								
Prepulse	Amplitude (nAm)		%PPI			Latency (ms)		
	Left	Right	Left		Right	Left		Right
Test-evoked								
Test alone	30.6±10.3	32.9±15.7				123.1±11.7		118.3±11.5
10ms	27.4±10.6	26.7±12.5	10.9±12.2		18.3±10.1	113.4±8.6		111.0±9.5
30ms	25.7±10.7	23.2±11.9	17.2±15.1		29.5±6.4	118.7±9.6		112.9±9.7
60ms	26.0±10.1	25.8±12.6	16.0±13.2		21.3±14.8	120.2±11.0		116.5±8.1
100ms	28.3±10.2	28.0±12.3	6.3±16.8		12.3±11.3	122.5±10.5		120.6±11.0
150ms	30.2±9.9	30.4±13.4	-0.1±17.6		7.6±15.1	124.9±11.6		120.2±9.4
200ms	30.3±10.1	32.6±15.6	1.1±11.9		1.1±16.0	122.2±9.6		117.8±7.7
Prepulse-evoked								
10ms	9.2±4.5	9.2±3.9						
30ms	9.4±3.2	8.9±3.7						
60ms	9.2±4.2	9.7±4.7						
100ms	9.0±3.5	8.8±3.2						
150ms	8.6±2.9	9.0±4.4						
200ms	8.7±3.2	7.4±3.9						
**Experiment 1–2**								
Test-evoked								
Test alone	25.8±12.8	26.9±12.8				117.4±11.4		107.6±6.7
300ms	26.1±16.3	28.3±12.9	2.0±20.0		-6.8±11.4	118.3±7.2		111.3±6.1
400ms	26.2±14.4	27.0±13.1	0.1±10.6		-1.5±14.3	116.9±13.3		110.4±6.2
500ms	25.1±15.1	27.4±15.2	5.4±15.7		1.4±16.5	119.7±11.3		111.6±6.4
600ms	23.8±12.6	24.7±13.4	8.2±11.8		10.8±15.2	117.9±10.6		108.3±9.0
700ms	25.3±13.3	25.6±14.1	2.7±9.3		8.4±14.6	119.3±12.2		106.5±8.9
800ms	25.7±14.3	25.9±12.4	2.1±12.7		3.1±10.8	120.6±11.3		109.2±6.9
Prepulse-evoked								
400ms	9.2±6.2	8.9±4.8				126.7±18.9		124.5±11.0
**Experiment 1–3**								
Test-evoked								
Test alone	29.3±18.4	29.0±13.6				116.0±14.6		112.5±12.8
300ms	26.1±17.5	28.6±13.3	11.1±25.3		-2.0±27.2	118.1±15.7		117.0±12.8
400ms	26.5±19.4	26.5±12.4	13.1±15.0		7.6±9.5	117.0±14.6		117.0±13.4
500ms	24.4±15.8	26.8±15.8	14.7±20.3		11.2±17.2	117.0±14.9		114.4±13.0
600ms	23.7±16.5	26.5±15.5	19.3±16.8		11.9±11.9	120.7±15.3		114.4±12.1
700ms	26.1±16.2	26.0±14.1	10.3±13.2		13.6±10.7	117.5±15.4		117.7±12.8
800ms	25.5±16.3	26.8±15.5	13.4±14.6		10.2±16.7	121.1±14.5		114.3±12.9
Prepulse-evoked								
400ms	16.1±10.7	18.5±11.0				124.8±10.6		131.1±10.0
**Experiment 1–4**								
Test-evoked								
Test alone	24.8±14.0	30.7±14.9				119.4±7.4		115.1±11.9
10ms	23.7±13.2	26.1±13.9	6.3±19.2		15.2±14.6	112.5±10.1		104.6±9.7
20ms	22.2±11.4	22.8±13.2	11.8±21.7		25.9±17.3	114.3±7.4		110.2±11.3
30ms	20.2±12.2	22.8±12.4	22.7±15.3		25.9±15.5	115.4±7.3		107.1±12.9
40ms	20.5±13.3	20.2±10.3	22.1±20.7		33.3±12.1	118.7±9.6		113.6±13.4
50ms	21.2±13.6	21.9±10.7	19.8±23.3		28.3±16.7	115.8±8.6		112.8±13.4
60ms	19.9±11.7	23.8±11.4	24.2±15.0		19.5±18.5	118.2±6.8		117.8±11.3
70ms	19.9±12.6	24.3±12.0	22.6±14.5		20.3±16.3	118.3±9.3		114.6±8.9
Prepulse-evoked								
10ms	8.2±6.4	9.9±5.4				135.5±28.4		140.0±18.1
20ms	9.4±4.6	10.3±4.8				135.0±32.0		140.0±18.9
30ms	10.8±7.5	9.9±5.9				141.2±34.8		137.7±27.1
40ms	10.1±5.7	11.3±5.4				144.2±29.3		144.1±27.5
50ms	9.5±5.2	10.9±4.5				148.5±26.7		143.8±22.7
60ms	9.5±5.4	10.4±4.7				132.1±23.6		139.0±23.2
70ms	11.3±5.8	10.7±4.9				136.2±17.7		130.4±21.8

**Table 2:** 

Experiment 2						
Prepulse	Amplitude (nAm)		%PPI		Latency (ms)	
	Left	Right	Left	Right	Left	Right
Test-evoked						
Test alone	31.9±13.6	34.0±16.2			122.9±9.5	118.5±13.3
1-Prepulse	21.7±13.2	23.3±13.7	34.7±16.4	31.7±11.5	121.7±7.9	115.1±11.4
2-Prepulse	15.4±9.6	16.8±8.4	52.2±19.8	46.8±20.4	117.3±12.4	114.6±11.5
3-Prepulse	11.2±4.8	10.1±5.9	62.3±17.8	68.9±12.4	113.2±14.6	109.5±15.8
4-Prepulse	9.4±5.4	7.9±4.6	69.2±16.7	71.7±22.3	114.4±17.2	109.9±16.2
6030 Prepulse	13.7±9.4	12.5±10.0	58.5±15.6	66.0±12.5	120.0±13.5	110.3±15.7
Prepulse-evoked						
1-Prepulse	8.2±5.0	10.0±4.4			132.0±15.5	127.1±15.2
2-Prepulse	9.9±5.3	12.4±6.8			136.2±18.1	132.4±10.6
3-Prepulse	11.7±7.8	14.4±8.2			133.7±18.0	138.8±11.3
4-Prepulse	12.9±7.9	14.5±6.5			128.8±12.7	138.0±15.1
6030 Prepulse	8.3±5.0	8.6±3.8			126.0±24.2	125.1±25.0

### Experiment 2

In order to confirm that inhibition was not an event within PC-PC excitatory transmission, but involved an active inhibitory process within microcircuits, the effects of prepulses with a train of clicks (Fig 3A) were examined in Experiment 2. Fig 3B shows the grand-averaged waveform of the Test- and Prepulse-evoked responses. The amplitude of the Test-evoked response was compared among five conditions including conditions without (Test alone) and with Prepulses of 1–4 clicks. A two-way ANOVA (Hemisphere X Prepulse) identified Prepulse (F_4,48_ = 35.08, p = 1.1 x 10^−13^, partial η^2^ = 0.75), but not Hemisphere (F_1,12_ = 0.07, p = 0.80) as a significant factor for the amplitude of the Test-evoked response. As shown in Fig 3B and 3C, the degree of inhibition increased with an increase in the number of Prepulse clicks, as expected. The amplitude of the Prepulse-evoked response also increased with higher click numbers, and the difference among the four Prepulses was significant (F_3,36_ = 11.16, p = 2.5 x 10^−5^, partial η^2^ = 0.48). When %PPI was plotted against the amplitude of the Prepulse-evoked response, their function was almost linear (Fig 3C, filled black circles). These results suggested that the degree of inhibition depended on the strength of the Prepulse; however, it remained unclear whether the amplitude of the Prepulse-evoked cortical response is important.

Therefore, we compared two Prepulses, one with clicks at 60 and 50 ms (6050 Prepulse) and another at 60 and 30 ms (6030 Prepulse) before the Test onset (Fig 3D). The results of Experiment 1 indicated that the second click at 30 ms of the 6030 Prepulse would be more strongly inhibited by the first click at 60 ms (i.e. the interval is 30 ms) than that at 50 ms of the 6050 Prepulse (10 ms interval) while the inhibitory ability on the Test response of each single click (at 30, 50, and 60 ms) would not differ significantly. As expected, the amplitude of the Prepulse-evoked cortical response was greater for the 6050 Prepulse (11.3 nAm) than the 6030 Prepulse (8.6 nAm)(Fig 3E). A statistical analysis (2 X 2 ANOVA) revealed a slight difference between the Prepulses (F_1,12_ = 4.39, p = 0.058, partial η^2^ = 0.27). On the other hand, as shown in Fig 3E, the Test-evoked response was more strongly inhibited by the 6030 Prepulse (62.3%) than the 6050 Prepulse (49.5%)(F_1,12_ = 6.59, p = 0.025, partial η^2^ = 0.36). When the amplitude and %PPI of the Prepulse-evoked response for the 6030 Prepulse were plotted in Fig 3C (orange filled circles), they appeared to be outside the regression line for the 1–4 Prepulses, suggesting that the degree of PPI did not depend on the amplitude of the Prepulse-evoked response. More importantly, these results indicated that the inhibitory ability of the Prepulse on the Test response was not suppressed when its excitatory response was suppressed by a prepulse.

## Discussion

The results of the present study showed that Change-N1m was clearly inhibited by a weak leading change stimulus and also that this inhibition reflected inhibitory actions on the generation of Change-N1m, but not an exclusive event within PC-PC excitatory synapses. Since Change-N1m used in the present study as the Test cortical response represents a kind of higher brain function to automatically detect changes in the sensory environment and to facilitate the execution of appropriate defensive behaviors [18], the present results indicated that several inhibitory mechanisms were engaged in this specific neural circuit. Given the role of change-related cortical responses as the automatic and early stage of defense reactions, its inhibition appears to function in order to temporally sharpen activation due to a salient event and to protect processing from being interfered with by subsequent events.

### Data interpretation

The results showing that the Prepulse at approximately the excitatory threshold suppressed the Test response indicated that inhibition cannot be explained by passive events such as the refractory period or fatigue. Although a depressing PC-PC synapse is one candidate for the inhibitory mechanism, weak activation is unlikely to be the main mechanism regardless of whether it is presynaptic [36] or postsynaptic [37] because the degree of depression in postsynaptic PCs is known to depend on the amplitude of EPSPs in presynaptic PCs [38]. The time course of inhibition in the present study was also not consistent with that of paired pulse or frequency-dependent synaptic depression in which suppression increased with an increase in input frequency [39], i.e. a shorter pulse interval caused greater suppression. The results of Experiment 2 showed that the inhibitory action of the Prepulse was not inhibited when its excitatory response was inhibited by a prepulse, which clearly indicated that the Prepulse-evoked response and Prepulse-evoked inhibition reflected activation in two distinct pathways. Therefore, the present results suggest that the threshold to activate the inhibitory pathway in the Change-N1m circuit was lower than or equal to that to activate the excitatory pathway, which is consistent with previous studies using intracellular recordings. For example, in a study using callosally-induced postsynaptic potentials evoked in PCs in the rat frontal cortex [29], the threshold intensity did not differ between EPSPs and IPSPs. Furthermore, an afferent has been shown to evoke larger EPSPs in inhibitory interneurons than in PCs (for a review, see [40]). These findings suggest that the activation of PCs is almost always followed by that of interneurons.

Since the Test and Prepulse have the same properties, they are expected to activate the same circuits. Therefore, low-threshold self-regulatory inhibition was considered to be a fundamental mechanism of control for the change-detection circuit in the present study. In order to evoke a clear MEG response such as Change-N1m in this study, a minimum of tens of thousands neurons have to be activated simultaneously [41]. By considering the strong inhibition of Change-N1m (e.g. 70% inhibition on average by the 4-Prepulse in Experiment 2), all these neurons were considered to be under inhibitory control. The underlying mechanism can be that each PC inhibits itself through a feedback pathway via an interneuron or that a PC that drives Change-N1m also activates an interneuron targeting Change-N1m-evoking PCs (feedforward). In either case, each interneuron needs to cover at least several PCs because interneurons comprise approximately 20% of the cortical population [42–44]. Therefore, each PC responsible for Change-N1m may be inhibited via nearby interneurons in a ‘blanket of inhibition’ manner [45], while the group of neurons for Change-N1m as a whole may be inhibited by thousands of ‘blankets’. The term ‘blanket of inhibition’ describes the dense and unspecific innervation of local PCs by GABA interneurons within restricted intralaminar territories of 200 μm radius. The manner seems as though the interneurons were extending a ‘blanket of inhibition’ [45]. Because the auditory cortex is outside the PPI pathway for conventional acoustic startle reflexes that is composed of the cochlear nucleus, caudal pontine reticular nucleus (giant neurons), and motoneurons [23,46], the present results may suggest that prepulse-induced suppression is not specific to startle pathways but represents a non-specific self-regulatory mechanism of sensory processing.

### Interneuron subclasses involved

Although non-invasive neuroimaging studies like the present study cannot reveal neural mechanisms in detail, some discussion can be made when the present results are compared with knowledge about interneurons obtained from whole-cell recording studies. Here we want to discuss the possible contribution of interneuron subclasses to the present PPI. Since the time course of inhibition was drawn using a weak prepulse of a single click at approximately the excitation threshold (Figs 2A and 3A), it can be regarded as mainly representing that of IPSPs not significantly confounded by changes in EPSPs in PC-PC excitatory transmission. The present results revealed the presence of several temporally distinct inhibitions. As for short-latency inhibition peaking at approximately 20–30 ms, its time course appeared to be consistent with that of classical GABA_A_-mediated IPSPs [29,30,47–50]. For example, the peak latency of early hyperpolarization evoked in PCs was previously reported to be 10–30 ms in the rat sensorimotor cortex following white matter stimulation [30], 29.4 ms in the rat frontal cortex following stimulation of the corpus callosum [29], 28 ms in the human temporal cortex following stimulation of the adjacent white matter [48], and earlier when interneurons were stimulated by a current injection [50]. Short-latency IPSPs are shortly preceded by EPSPs and typically outlast them [47], suggesting that short-latency IPSPs play a role in generating synchronized, transient activation in target PCs. An *in vivo* whole-cell recording study in the mouse auditory cortex demonstrated that such a precise and stereotyped sequence of excitation and inhibition made the PC fire within a few milliseconds in response to auditory stimuli [51]. Previous studies reported that several different GABAergic neurons evoked similar short-latency IPSPs (e.g. [50]). Of these, several lines of evidence indicated that fast spiking (FS) or parvalbumin-positive (PV) interneurons [3], the largest subclass of interneurons [3,52], played an important role in fast and transient inhibition (for review, see [53]). PV interneurons are known to be densely connected to nearby PCs in a non-selective manner across cortical areas and layers, suggesting their role in the unspecific blanket of inhibition [54], which also appears consistent with the present inhibition. In the mouse primary auditory cortex, PV interneurons are well tuned for frequency, which suggests that they are driven nonselectively by the local network [55].

Long-latency inhibition at approximately 600 ms differed from short-latency inhibition in late-onset, long duration, and weak inhibition. A candidate responsible for late inhibition is a somatostatin (SOM)-positive interneuron, which predominantly is a Martinotti cell [2,56]. Its inhibition has been shown to increase with higher frequencies and numbers of APs in presynaptic PCs [31,57,58]. Due to their dependency on the activity of presynaptic PCs, the amplitude and latency of IPSPs markedly vary according to excitatory inputs. For example, in a study by Silberberg and Markram [31], a train of 15 APs at 40 Hz applied to a presynaptic PC evoked a weak IPSP in a target PC at 300–500 ms (see Fig 3A). As the latency is clearly longer with an AP train of a lower frequency, this inhibitory mechanism appears to be the most suitable explanation for the very late inhibition observed in the present study in terms of response latency based on the weak Prepulse around the excitation threshold.

SOM interneurons were previously reported to make unspecific dense connections with neighboring PCs, and thus, are considered to provide a ‘blanket of inhibition’ [59] similar to PV interneurons as described above. Thus, two similar but temporally distinct local inhibitions may be involved. They may work complementarily in time or in different presynaptic discharge patterns. Short-term synaptic plasticity has also been shown to differ among interneurons, thereby supporting their different roles (for a review, see [60]). For example, a study by Beierlein et al. on PV and SOM neurons in the rat barrel cortex [61] demonstrated that excitatory inputs from PCs to PV neurons and inhibitory inputs from PV neurons to PCs both caused rapidly decreasing responses to a train of APs. Thus, PV synapses are tuned for reliability in response to a transient activation. On the other hand, PC-SOM synapses displayed a marked short-term facilitation, while SOM-evoked IPSPs slightly facilitated or moderately depressed, which yielded an inhibitory system that was silent to a transient input, but became increasingly responsive to sustained high-frequency activity. Thus, these findings support the complementary roles of PV and SOM neurons in controlling cortical network excitability. In the Change-N1m circuit, the former appeared to produce sharpened, synchronized firing in PCs, while the latter may be involved in the suppression of excessive activity or in synaptic memory.

### Limitations

The present results implied the presence of a second short-latency inhibition 20–30 ms after the first one (Figs 2D and 3E). We attributed the stronger inhibition of the 6030 Prepulse than the 6050 Prepulse in Experiment 2 (Fig 3E) to the summation of two temporally distinct inhibitions. There are several candidates such as GABA_B_-mediated inhibition [47,49], and GABA_A_ and GABA_B_ receptor-mediated inhibition by neurogliaform neurons [62]. However, their dynamics do not appear to coincide exactly with our results. The present study did not provide direct evidence regarding the subclass of interneurons involved in auditory PPI. In addition, there also remains the possibility that other mechanisms such as the depressing synapse contribute to PPI. Future studies such those in which GABAergic drugs are administered to human subjects may extend our understanding; however, there are limitations to studies in humans. The site of inhibition remains to be clarified. However, previous studies using the auditory brainstem response showed that brain areas higher than the midbrain were responsible for producing change-related brain responses [63,64]. Therefore, the thalamus or auditory cortex is the target site of inhibition, and this should be confirmed by further studies. Another limitation of the present study is that we could not clarify gender differences. Because significant gender differences are reported in studies using PPI of startle reflexes [65,66], there might be differences in the present study, although the underlying neural mechanisms may also be different between the present and conventional PPI.

## Conclusion

The present results showed that, in the Change-N1m circuit, excitation and inhibition always coexisted, thereby supporting the importance of interneurons to process sensory information. However, this also indicates the difficulties associated with human studies without methods such as voltage-clamping to observe these events separately. Even with such limitations, the present method appeared to be useful for evaluating inhibitory functions in each individual. Since the inhibitory system is similar at least in several respects across layers, cortical areas, and species (e.g. [47,48,54,67]), the present inhibition in a specific circuit may represent the fundamental inhibitory functions of several subclasses of interneurons. Since many diseases are considered to have deficits in the inhibitory system, the present method provides an insight into the pathophysiologies of these diseases. For example, patients with schizophrenia may have disrupted PV circuits [12,68], which may be confirmed by abnormalities in the short-latency inhibition of the present study.

## References

[1] DeFelipe J. Neocortical neuronal diversity: chemical heterogeneity revealed by colocalization studies of classic neurotransmitters, neuropeptides, calcium-binding proteins, and cell surface molecules. Cereb Cortex 1993;3:273–289. 8104567 10.1093/cercor/3.4.273

[2] Kawaguchi Y, Kubota Y. Physiological and morphological identification of somatostatin- or vasoactive intestinal polypeptide-containing cells among GABAergic cell subtypes in rat frontal cortex. J Neurosci. 1996;16:2701–2715. 8786446 10.1523/JNEUROSCI.16-08-02701.1996 PMC6578756

[3] Kawaguchi Y, Kubota Y. GABAergic cell subtypes and their synaptic connections in rat frontal cortex. Cereb Cortex 1997;7:476–486. 9276173 10.1093/cercor/7.6.476

[4] Thomson AM, Deuchars J. Synaptic interactions in neocortical local circuits: dual intracellular recordings in vitro. Cereb Cortex 1997;7:510–522. 9276176 10.1093/cercor/7.6.510

[5] Somogyi P, Tamás G, Lujan R, Buhl EH. Salient features of synaptic organisation in the cerebral cortex. Brain Res Brain Res Rev. 1998;26:113–135. 9651498 10.1016/s0165-0173(97)00061-1

[6] Gupta A, Wang Y, Markram H. Organizing principles for a diversity of GABAergic interneurons and synapses in the neocortex. Science 2000;287:273–278. 10634775 10.1126/science.287.5451.273

[7] Markram H, Toledo-Rodriguez M, Wang Y, Gupta A, Silberberg G, Wu C. Interneurons of the neocortical inhibitory system. Nat Rev Neurosci. 2004;5:793–807. 15378039 10.1038/nrn1519

[8] DeFelipe J, López-Cruz PL, Benavides-Piccione R, Bielza C, Larrañaga P, Anderson S, et al New insights into the classification and nomenclature of cortical GABAergic interneurons. Nat Rev Neurosci. 2013;14:202–216. 10.1038/nrn3444 23385869 PMC3619199

[9] Hu H, Gan J, Jonas P. Fast-spiking, parvalbumin^+^ GABAergic interneurons: from cellular design to microcircuit function. Science 2014;345:1255263 10.1126/science.1255263 25082707

[10] McCormick DA, Contreras D. On the cellular and network bases of epileptic seizures. Annu Rev Physiol. 2001;63:815–846. 11181977 10.1146/annurev.physiol.63.1.815

[11] Noebels JL. The biology of epilepsy genes. Annu Rev Neurosci. 2003;26:599–625. 14527270 10.1146/annurev.neuro.26.010302.081210

[12] Marín O. Interneuron dysfunction in psychiatric disorders. Nat Rev Neurosci. 2012; 13:107–120. 10.1038/nrn3155 22251963

[13] Pehrson AL, Sanchez C. Altered γ-aminobutyric acid neurotransmission in major depressive disorder: a critical review of the supporting evidence and the influence of serotonergic antidepressants. Drug Des Devel Ther. 2015;9:603–624. 10.2147/DDDT.S62912 25653499 PMC4307650

[14] Emrich HM, von Zerssen D, Kissling W, Möller HJ, Windorfer A. Effect of sodium valproate on mania. The GABA-hypothesis of affective disorders. Arch Psychiatr Nervenkr. 1980;229:1–16. 6778456 10.1007/BF00343800

[15] Möhler H. The GABA system in anxiety and depression and its therapeutic potential. Neuropharmacology 2012;62:42–53. 10.1016/j.neuropharm.2011.08.040 21889518

[16] Cellot G, Cherubini E. GABAergic signaling as therapeutic target for autism spectrum disorders. Front Pediatr. 2014;2:70 10.3389/fped.2014.00070 25072038 PMC4085902

[17] Gironell A. The GABA hypothesis in essential tremor: lights and shadows. Tremor Other Hyperkinet Mov. 2014;4:254. 10.7916/D8SF2T9C PMC4108714 25120944

[18] Inui K, Tsuruhara A, Kodaira M, Motomura E, Tanii H, Nishihara M, et al Prepulse inhibition of auditory change-related cortical responses. BMC Neurosci. 2012;13:135 10.1186/1471-2202-13-135 23113968 PMC3502566

[19] Inui K, Tsuruhara A, Nakagawa K, Nishihara M, Kodaira M, Motomura E, et al Prepulse inhibition of change-related P50m no correlation with P50m gating. Springerplus 2013;2:588 10.1186/2193-1801-2-588 24255871 PMC3825222

[20] Graham FK. Presidential Address: The more or less startling effects of weak prestimulation. Psychophysiology 1975;12:238–248. 1153628 10.1111/j.1469-8986.1975.tb01284.x

[21] Braff DL, Geyer MA, Swerdlow NR. Human studies of prepulse inhibition of startle: normal subjects, patient groups, and pharmacological studies. Psychopharmacology 2001;156:234–258. 11549226 10.1007/s002130100810

[22] Swerdlow NR, Weber M, Qu Y, Light GA, Braff DL. Realistic expectations of prepulse inhibition in translational models for schizophrenia research. Psychopharmacology 2008;199:331–388. 10.1007/s00213-008-1072-4 18568339 PMC2771731

[23] Swerdlow NR, Geyer MA, Braff DL. Neural circuit regulation of prepulse inhibition of startle in the rat: current knowledge and future challenges. Psychopharmacology 2001;156:194–215. 11549223 10.1007/s002130100799

[24] Inui K, Urakawa T, Yamashiro K, Otsuru N, Nishihara M, Takeshima Y, et al Non-linear laws of echoic memory and auditory change detection in humans. BMC Neurosci. 2010;11:80 10.1186/1471-2202-11-80 20598152 PMC2904354

[25] Swerdlow NR, Talledo J, Shoemaker JM, Codon K, Goins J, Auerbach PP. Weak prepulses inhibit but do not elicit startle in rats and humans. Biol Psychiatry. 2004;55:1195–1198. 15184039 10.1016/j.biopsych.2004.02.030

[26] Otsuru N, Tsuruhara A, Motomura E, Tanii H, Nishihara M, Inui K, et al Effects of acute nicotine on auditory change-related cortical responses. Psychopharmacology 2012;224:327–335. 10.1007/s00213-012-2757-2 22707251

[27] Kodaira M, Tsuruhara A, Motomura E, Tanii H, Inui K, Kakigi R. Effects of acute nicotine on prepulse inhibition of auditory change-related cortical responses. Behav Brain Res. 2013;256:27–35. 10.1016/j.bbr.2013.07.045 23933145

[28] Vogels TP, Abbott LF. Gating multiple signals through detailed balance of excitation and inhibition in spiking networks. Nat Neurosci. 2009;12:483–491. 10.1038/nn.2276 19305402 PMC2693069

[29] Kawaguchi Y. Receptor subtypes involved in callosally-induced postsynaptic potentials in rat frontal agranular cortex in vitro. Exp Brain Res. 1992;88:33–40. 1347272 10.1007/BF02259126

[30] Avoli M. Inhibitory potentials in neurons of the deep layers of the in vitro neocortical slice. Brain Res. 1986;370:165–170. 3011189 10.1016/0006-8993(86)91118-2

[31] Silberberg G, Markram H. Disynaptic inhibition between neocortical pyramidal cells mediated by Martinotti cells. Neuron 2007;53:735–746. 17329212 10.1016/j.neuron.2007.02.012

[32] Inui K, Urakawa T, Yamashiro K, Otsuru N, Takeshima Y, Nishihara M, et al Echoic memory of a single pure tone indexed by change-related brain activity. BMC Neurosci. 2010; 11:135 10.1186/1471-2202-11-135 20961454 PMC2978218

[33] Nakagawa K, Otsuru N, Inui K, Kakigi R. Change-related auditory P50: A MEG study. NeuroImage 2014;86:131–137. 10.1016/j.neuroimage.2013.07.082 23933044

[34] Yamashiro K, Inui K, Otsuru N, Kakigi R. Change-related responses in the human auditory cortex: an MEG study. Psychophysiology 2011;48:23–30. 10.1111/j.1469-8986.2010.01038.x 20525009

[35] Inui K, Okamoto H, Miki K, Gunji A, Kakigi R. Serial and parallel processing in the human auditory cortex: a magnetoencephalographic study. Cereb Cortex 2006;16:18–30. 15800024 10.1093/cercor/bhi080

[36] Galarreta M, Hestrin S. Frequency-dependent synaptic depression and the balance of excitation and inhibition in the neocortex. Nat Neurosci. 1998;1:587–594. 10196566 10.1038/2822

[37] Trussell LO, Zhang S, Raman IM. Desensitization of AMPA receptors upon multiquantal neurotransmitter release. Neuron 1993;10:1185–1196. 7686382 10.1016/0896-6273(93)90066-z

[38] Thomson AM, Deuchars J, West DC. Large, deep layer pyramid-pyramid single axon EPSPs in slices of rat motor cortex display paired pulse and frequency-dependent depression, mediated presynaptically and self-facilitation, mediated postsynaptically. J Neurophysiol. 1993;70:2354–2369. 8120587 10.1152/jn.1993.70.6.2354

[39] Thomson AM. Activity-dependent properties of synaptic transmission at two classes of connections made by rat neocortical pyramidal axons in vitro. J Physiol. 1997; 502: 131–147. 9234202 10.1111/j.1469-7793.1997.131bl.x PMC1159577

[40] Isaacson JS, Scanziani M. How inhibition shapes cortical activity. Neuron 2011;72:231–243. 10.1016/j.neuron.2011.09.027 22017986 PMC3236361

[41] Okada Y. Neurogenesis of evoked magnetic fields In: Williamson SH, Romani GL, Kaufman L, Modena I, editors. Biomagnetism: an Interdisciplinary Approach. New York: Plenum Press; 1983 pp. 399–408.

[42] Meinecke DL, Peters A. GABA immunoreactive neurons in rat visual cortex. J Comp Neurol. 1987;261:388–404. 3301920 10.1002/cne.902610305

[43] DeFelipe J, Alonso-Nanclares L, Arellano JI. Microstructure of the neocortex: comparative aspects. J Neurocytol. 2002;31:299–316. 12815249 10.1023/a:1024130211265

[44] Meyer HS, Schwarz D, Wimmer VC, Schmitt AC, Kerr JN, Sakmann B, et al Inhibitory interneurons in a cortical column form hot zones of inhibition in layers 2 and 5A. Proc Natl Acad Sci U S A. 2011;108:16807–16812. 10.1073/pnas.1113648108 21949377 PMC3189020

[45] Karnani MM, Agetsuma M, Yuste R. A blanket of inhibition: functional inferences from dense inhibitory connectivity. Curr Opin Neurobiol. 2014;26:96–102. 10.1016/j.conb.2013.12.015 24440415 PMC4024353

[46] Fendt M, Li L, Yeomans JS. Brain stem circuits mediating prepulse inhibition of the startle reflex. Psychopharmacology 2001;156:216–224. 11549224 10.1007/s002130100794

[47] Connors BW, Malenka RC, Silva LR. Two inhibitory postsynaptic potentials, and GABAA and GABAB receptor-mediated responses in neocortex of rat and cat. J Physiol. 1988;406:443–468. 2855437 10.1113/jphysiol.1988.sp017390 PMC1191109

[48] McCormick DA. GABA as an inhibitory neurotransmitter in human cerebral cortex. J Neurophysiol. 1989;62:1018–1027. 2573696 10.1152/jn.1989.62.5.1018

[49] Benardo LS. Separate activation of fast and slow inhibitory postsynaptic potentials in rat neocortex in vitro. J Physiol. 1994;476:203–215. 7913968 10.1113/jphysiol.1994.sp020124 PMC1160434

[50] Tamás G, Buhl EH, Somogyi P. Fast IPSPs elicited via multiple synaptic release sites by different types of GABAergic neurone in the cat visual cortex. J Physiol. 1997; 500:715–738. 9161987 10.1113/jphysiol.1997.sp022054 PMC1159420

[51] Wehr M, Zador AM. Balanced inhibition underlies tuning and sharpens spike timing in auditory cortex. Nature 2003;426:442–446. 14647382 10.1038/nature02116

[52] Gonchar Y, Burkhalter A. Three distinct families of GABAergic neurons in rat visual cortex. Cereb Cortex 1997;7:347–358. 9177765 10.1093/cercor/7.4.347

[53] Fino E, Packer AM, Yuste R. The logic of inhibitory connectivity in the neocortex. Neuroscientist 2013;19:228–237. 10.1177/1073858412456743 22922685 PMC4133777

[54] Packer AM, Yuste R. Dense, unspecific connectivity of neocortical parvalbumin-positive interneurons: a canonical microcircuit for inhibition? J Neurosci. 2011;31:13260–13271. 10.1523/JNEUROSCI.3131-11.2011 21917809 PMC3178964

[55] Moore AK, Wehr M. Parvalbumin-expressing inhibitory interneurons in auditory cortex are well-tuned for frequency. J Neurosci. 2013;33:13713–13723. 10.1523/JNEUROSCI.0663-13.2013 23966693 PMC3755717

[56] McGarry LM, Packer AM, Fino E, Nikolenko V, Sippy T, Yuste R. Quantitative classification of somatostatin-positive neocortical interneurons identifies three interneuron subtypes. Front Neural Circuits 2010;4:12 10.3389/fncir.2010.00012 20617186 PMC2896209

[57] Kapfer C, Glickfeld LL, Atallah BV, Scanziani M. Supralinear increase of recurrent inhibition during sparse activity in the somatosensory cortex. Nat Neurosci. 2007; 10:743–753. 17515899 10.1038/nn1909 PMC3518866

[58] Berger TK, Silberberg G, Perin R, Markram H. Brief bursts self-inhibit and correlate the pyramidal network. PLoS Biol. 2010;8: e1000473 10.1371/journal.pbio.1000473 20838653 PMC2935452

[59] Fino E, Yuste R. Dense inhibitory connectivity in neocortex. Neuron 2011;69:1188–1203. 10.1016/j.neuron.2011.02.025 21435562 PMC3086675

[60] Thomson AM, Lamy C. Functional maps of neocortical local circuitry. Front Neurosci. 2007; 1:19–42. 10.3389/neuro.01.1.1.002.2007 18982117 PMC2518047

[61] Beierlein M, Gibson JR, Connors BW. Two dynamically distinct inhibitory networks in layer 4 of the neocortex. J Neurophysiol. 2003; 90:2987–3000. 12815025 10.1152/jn.00283.2003

[62] Tamás G, Lőrincz A, Simon A, Szabadics J. Identified sources and targets of slow inhibition in the neocortex. Science 2003;299:1902–1905. 12649485 10.1126/science.1082053

[63] Nishihara M, Inui K, Morita T, Kodaira M, Mochizuki H, Otsuru N, et al Echoic memory: investigation of its temporal resolution by auditory offset cortical responses. PLoS One. 2014;9:e106553 10.1371/journal.pone.0106553 25170608 PMC4149571

[64] Tanahashi M, Motomura E, Inui K, Ohoyama K, Tanii H, Konishi Y, et al Auditory change-related cerebral responses and personality traits. Neurosci Res. 2016 10.1016/j.neures.2015.08.005 26360233

[65] Kofler M, Kumru H, Schaller J, Saltuari L. Blink reflex prepulse inhibition and excitability recovery: influence of age and sex. Clin Neurophysiol. 2013;124:126–35. 10.1016/j.clinph.2012.07.001 22857876

[66] Kumari V. Sex differences and hormonal influences in human sensorimotor gating: implications for schizophrenia. Curr Top Behav Neurosci. 2011;8:141–154. 10.1007/7854_2010_117 21374020

[67] Kätzel D, Zemelman BV, Buetfering C, Wölfel M, Miesenböck G. The columnar and laminar organization of inhibitory connections to neocortical excitatory cells. Nat Neurosci. 2011;14:100–107. 10.1038/nn.2687 21076426 PMC3011044

[68] Lewis DA. Inhibitory neurons in human cortical circuits: substrate for cognitive dysfunction in schizophrenia. Curr Opin Neurobiol. 2014;26:22–26. 10.1016/j.conb.2013.11.003 24650500 PMC4024332

